# Hypertension in a Patient With Polycystic Kidney Disease Complicated by Concomitant Pheochromocytoma

**DOI:** 10.1016/j.aace.2024.04.001

**Published:** 2024-04-13

**Authors:** Adelina Ameti, Peter A. Kopp, Nelly Pitteloud, Grégoire Wuerzner, Eric Grouzmann, Maurice Matter, Faiza Lamine, Olivier Phan

**Affiliations:** 1 Division of Endocrinology, Diabetes & Metabolism, Centre Hospitalier Universitaire Vaudois (CHUV), Lausanne, Switzerland; 2 Division of Nephrology and Hypertension, Centre Hospitalier Universitaire Vaudois (CHUV), Lausanne, Switzerland; 3 Division of Clinical Pharmacology, Catecholamine and Peptides Laboratory, CHUV, Lausanne, Switzerland; 4 Division of Visceral Surgery, University Hospital CHUV, Lausanne, Switzerland

**Keywords:** autosomal dominant polycystic disease, hypertension, pheochromocytoma, catecholamines, metanephrines

## Abstract

**Background:**

Due to the high prevalence of hypertension in patients with autosomal dominant polycystic kidney disease (ADPKD) and advanced chronic kidney disease, diagnosing secondary hypertension poses challenges. We present a rare case of pheochromocytoma in an ADPKD patient to highlight the diagnostic difficulties in identifying secondary hypertension due to pheochromocytoma/paraganglioma (PPGL) in end-stage renal disease (ESRD) patients.

**Case Report:**

A 48-year-old female with ADPKD and ESRD experienced recurrent hypertensive crises (up to 220/135 mmHg) accompanied by palpitations and tremors that recurred over the past 2 years. Introduction of a betablocker to the antihypertensive therapy aggravated her symptoms. The initial documentation of elevated urinary metanephrines was interpreted as false positive finding due to renal failure. Subsequent measurements of free plasma metanephrines revealed significant elevations raising suspicion of PPGL. Magnetic resonance imaging identified a 29 mm right adrenal mass. The patient underwent right adrenalectomy resulting in resolution of the hypertensive crises.

**Discussion:**

The diagnosis of PPGLs can present significant challenges and is further complicated in ESRD due to nonspecific clinical symptoms and diagnostic pitfalls. Less than 20 PPGL cases have been reported in patients with ESRD. The intolerance of beta-blocker therapy, as well as the use of a scoring system for the likelihood of PPGL should have raised suspicion.

**Conclusion:**

PPGL should be considered in all patients with uncontrolled hypertension and beta-blockers intolerance, even in the presence of other etiologic mechanisms such as ESRD. Measuring free plasma metanephrines provides the most reliable biochemical screening in the context of impaired renal function.

**Highlights**

• Pheochromocytoma can be a life-threatening condition.
• Hypertensive crises suggest pheochromocytoma even in patients with ADPKD.
• Beta-blocker intolerance: a key sign of potential pheochromocytoma.
• Plasma free metanephrines is the most reliable test in renal impairment.

**Clinical Relevance**

A 48-year-old female with autosomal dominant polycystic kidney disease and end-stage chronic kidney disease presented with recurrent hypertensive crises which ultimately led to the diagnosis of pheochromocytoma. The intricacies of interpreting plasma metanephrines and catecholamine tests in patients with advanced kidney disease (hemodialysis and end-stage renal failure) are discussed.

## Introduction

Autosomal dominant polycystic kidney disease (ADPKD) is a common hereditary disorder characterized by renal and extrarenal cyst formation. It is a leading cause of end-stage renal disease (ESRD).^1^

Renal disease is a well-known cause of secondary hypertension.^2^ The prevalence of hypertension among patients with ADPKD is approximately 70% to 80%, and in 60% it is present even before functional renal impairment.^3^ Mechanistically, the relationship between structural abnormalities and hypertension in ADPKD suggests that activation of the renin-angiotensin-aldosterone system play an important etiological role.^4^^,^^5^

Pheochromocytomas and secretory paragangliomas (PPGLs) are rare and treatable causes of secondary hypertension, with an incidence of about 0.6 cases per 100 000 person-years.^6^ To our knowledge, only 18 cases of PPGLs have been reported in patients with ESRD.^7^^,^^8^ Clinical signs and symptoms are heterogeneous and unspecific (hypertension, spells with palpitations, headaches, tremor, sweating, anxiety), and also dependent on the type of the secreted catecholamine(s).^9^ The diagnosis relies on biochemical testing documenting catecholamine excess and imaging studies to localize the adrenal or extra-adrenal neoplasm(s).^10^ In the context of renal failure, the diagnosis of PPGLs can be challenging due to unreliable urinary metanephrines measurements and increased levels of sulphated plasma metanephrines, which are normally cleared by the kidneys.^11^ Undiagnosed, PPGLs can be life-threatening,^6^ and lead to significant cardiovascular comorbidities.^12^ To illustrate the challenges in diagnosing secondary hypertension associated with PPGLs in patients with ESRD, we report a patient with ADPKD with hypertensive crises who was diagnosed with a pheochromocytoma.

## Case Report

A 48-year-old woman with ADPKD, caused by a pathogenic variant in the PKD1 gene, with end-stage chronic kidney disease (KDIGO G5A2 with preserved diuresis), has been referred to our tertiary center for the further evaluation of hypertensive crises. The patient reported a history of hypertension for the past 12 years. During her 3 pregnancies, she had pre-eclampsia. Her father, her brother, and her sister are also affected by ADPKD, but there was no family history of neuroendocrine tumors. The hypertensive crises, with values up to 220/135 mmHg, were first noticed at about age 46 years, increased in frequency over time, and were accompanied by palpitations and tremors. They lasted about 15 minutes and were followed by bradycardia (45 bpm), nausea, and dyspnea.

Her treatment consisted of candesartan 8 mg/day, hydrochlorothiazide 12.5 mg/d, and carvedilol 2 mg/d. Two years ago, she had been briefly treated with the unselective beta-blocker propranolol for the control of palpitations. A few weeks later, this treatment was discontinued because of increased palpitations, dyspnea, and flushing. The patient was switched to carvedilol and the dose was progressively decreased to a compounded preparation of 2 mg, which was the only tolerable dose for beta-blocker treatment.

One year before presentation to our clinic, urinary metanephrines measured elsewhere showed slightly increased metanephrine (1.7-fold increase) and normetanephrine levels (1.2-fold increase). As documented in the medical record, these findings were considered as false positive in the context of renal insufficiency affecting 24-hour urine volume determination, and normalization of the metanephrines by creatininuria. Biochemical testing in our center documented increased urinary metanephrines (metanephrine 4795 nmol/24 h; upper reference limit 1880 nmol/24 h, 2.55-fold increase), plasma free metanephrine (2.03 nmol/l; reference 0.03-0.85 nmol/l 2.38-fold increase), and plasma free normetanephrine (4.24 nmol/l: reference 0.04-1.39 nmol/l, 3-fold increase) (Table).

**Table:** Relevant Laboratory Results at Diagnosis and 6 Weeks After Right Adrenalectomy and Bilateral Nephrectomy

Laboratory	At diagnosis	Six weeks after surgery	Reference intervals in hypertensive patients^12^
Serum creatinine (μmol/l)	432	NA (Dialysis)	44-80
Urinary metanephrine (nmol/24 h)	4795	NA	<1880
Urinary normetanephrine (nmol/24 h)	NA	NA	<3800
Urinary methoxyramine (nmol/24 h)	503	NA	<1900
Plasma free metanephrine (nmol/l)	2.03	0.08	0.03-0.85
Plasma free normetanephrine (nmol/l)	4.24	0.23	0.04-1.39
Plasma free methoxyramine (nmol/l)	0.11	0.04	<0.06
Total plasma metanephrine (nmol/l)	362.0	18.09	0.66-13.45
Total plasma normetanephrine (nmol/l)	364.12	44.81	2.14-36.65
Total plasma methoxyramine (nmol/l)	28.01	24.17	0.59-4.19

Subsequent abdominal magnetic resonance imaging (MRI) revealed the presence of a 29 mm right adrenal mass, and a Ga-68-DOTATATE positron emission tomography-computed tomography did not show any other lesion suggestive for a PPGL (Fig.). Retrospective analysis of an MRI performed 2 years earlier revealed that the pheochromocytoma was present already at that time. However, it had been overlooked because of the multiple voluminous renal and hepatic cysts. In preparation for the surgical treatment, alpha-blockade with doxazosin, initially with 4 mg/d followed by a gradual increase to 16 mg/day, was initiated to achieve a blood pressure target <130/90 mmHg. This resulted in prompt control of the hypertensive crises and the associated spells after 3 weeks. Six weeks later, the patient underwent a right adrenalectomy and bilateral nephrectomy. The decision to perform bilateral nephrectomy was made based on the large size of the kidneys with voluminous renal cysts, and the preparation for renal transplantation as a candidate for a living donor transplant. No hemodynamic instability was observed during the procedure. Hemodialysis (HD) was initiated postsurgery and metanephrines measured 6 weeks later showed normal levels (Table).

**Fig:**
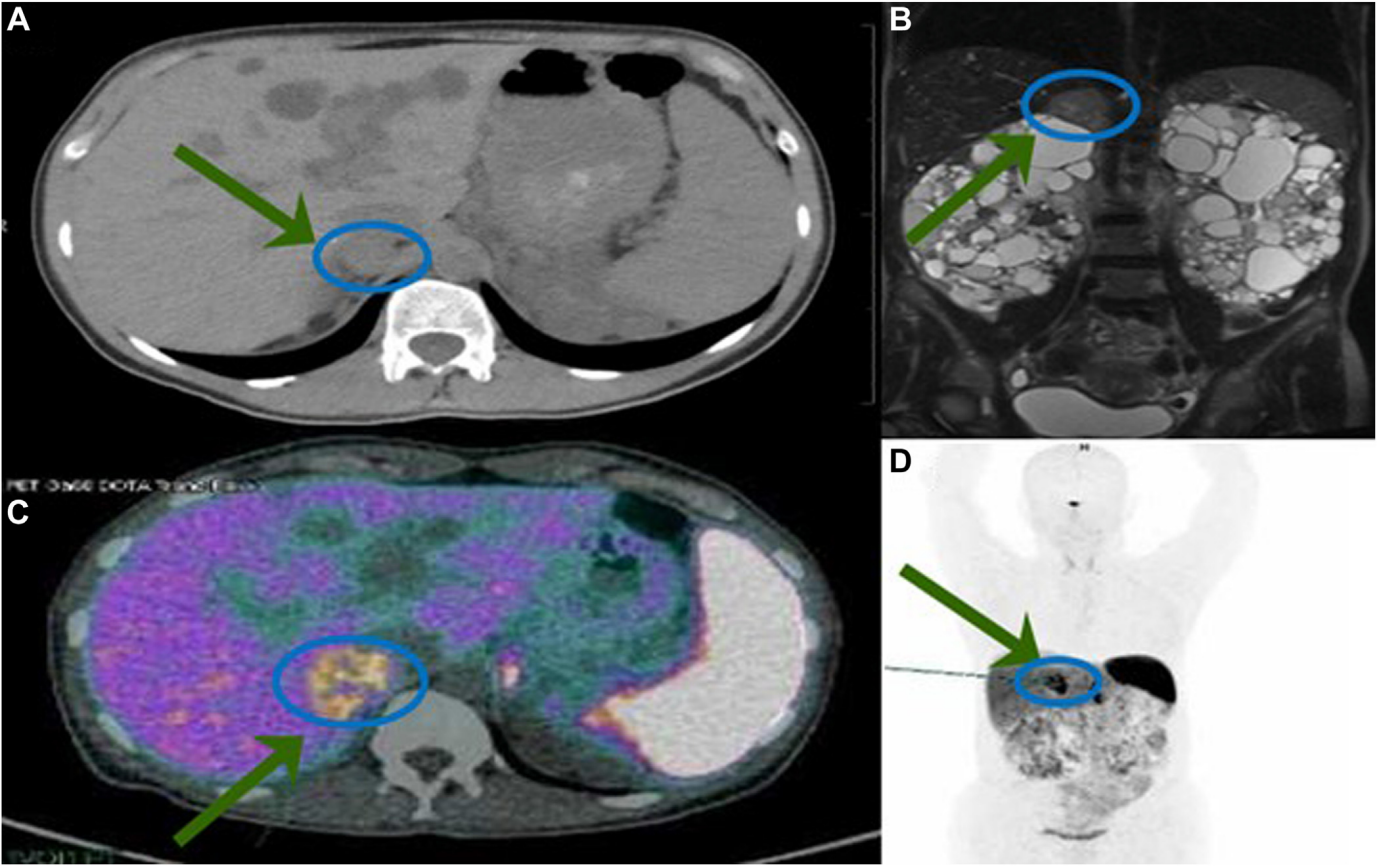
Imaging studies. (*A*) Abdominal computed tomography (CT). An adrenal tumor was present 2 years before diagnosis but overlooked because of the multiple, in part hemorrhagic and closely adjacent cysts in the liver and the right kidney. (*B*) Magnetic resonance imaging (MRI) at diagnosis documenting a 29 mm right adrenal lesion. (*C*, *D*) 86-Ga-DOTATATE positron emission tomography (PET)-CT demonstrating right adrenal uptake without other pathologic lesions.

Histopathology showed a well-delimited pheochromocytoma without lymphovascular invasion, the kidneys showed the characteristic alterations of PKD. The Pheochromocytoma of the Adrenal gland Scaled Score was 1.^13^ The tumor cells strongly expressed chromogranin A, synaptophysin and succinate dehydrogenase B. The Ki67 proliferative index was 1.5%. Exome sequencing for germline mutations associated with familial PPGLs was negative in the tested genes (VHL, SDHA, SDHB, SDHC, SDHD, FH, RET, NF1, MAX, TMEM27).^10^^,^^14^

## Discussion

The patient presented here developed hypertension in the context of PKD and ESRD, which was then complicated by secondary hypertension due to a pheochromocytoma. The diagnosis of PPGLs is challenging in the general population and even more complex in patients with ESRD and HD due to unspecific clinical signs and several pitfalls in measuring metanephrines. In a prospective study, the most frequent clinical sign was palpitations affecting 65% of patients, whereas office measurements of blood pressure and the presence of hypertension were not helpful for the diagnosis of PPGLs.^9^^,^^10^ A scoring system to predict the probability of a PPGL has been proposed for screening.^9^^,^^10^ It includes presenting signs and symptoms such as pallor, hyperhidrosis, tremor, palpitations, nausea, as well as heart rate and body mass index. Based on this score, the patient presented here had a high likelihood of PPGL (4 points; high clinical feature score defined as >3). Moreover, the intolerance to beta-blocker therapy reported by the patient should raise suspicion. Together with angiotensin receptor blockers and angiotensin-converting enzyme inhibitors, beta-blockers play a key role in the treatment of hypertension in ADPKD.^15^ However, beta-blockers can induce hypertensive crises and/or spells in patients with undiagnosed PPGLs due to an unopposed alpha-adrenergic effect of catecholamines; for this reason, it is recommended to first initiate therapy with an alpha-blocker in PPGL patients.^16^

In the context of renal failure, 24-hour urinary metanephrine and total metanephrine measurements are unreliable due to their dependence on renal function;^17^ in contrast, free plasma metanephrines can be used even in the setting of renal impairment as they are relatively independent of renal clearance.^11^ However, the results need to be interpreted cautiously because the free plasma metanephrine concentration may be increased secondary to sympathetic activation and an increase of catecholamine secretion from nerve terminals, as well as O-methylation in extra-neuronal tissues.^18^ HD may affect metanephrine measurements, and it has been suggested that (even) free plasma metanephrines are elevated in hemodialyzed patients (described in a small series as more than 50% above the upper limit of normal).^11^^,^^19^ The mechanism behind this elevation of metanephrines in HD patients remains unclear but may be related to impaired clearance and chronic sympathetic activation associated with the disease.^20^ A prospective study investigated the timing of metanephrine measurement during HD and showed that collecting a sample from the shunt in the last hour of dialysis reduced false positives.^20^ Lastly, as illustrated by the patient presented here, distinguishing adrenal neoplasms from hemorrhagic cysts on CT or MRI can be challenging in patients with PKD, and they can be overlooked unless there is a heightened degree of suspicion.

## Conclusion

PPGLs can be life-threatening because of the impact of catecholamines on the cardiovascular system. It is paramount to consider the possibility of a PPGL in all patients with uncontrolled hypertension and intolerance to beta-blockers, even if there are other concomitant etiologic mechanisms leading to hypertension as in this patient with ESRD due to ADPKD. The most reliable screening test in the setting of impaired renal function and ESRD is the measurement of free plasma metanephrines.

## Disclosure

The authors have no multiplicity of interest to disclose.
